# A cross-sectional study of alcohol consumption and alcoholic liver disease in Beijing: based on 74,998 community residents

**DOI:** 10.1186/s12889-022-13175-z

**Published:** 2022-04-12

**Authors:** Huai Wang, Pei Gao, Weixin Chen, Qianli Yuan, Min Lv, Shuang Bai, Jiang Wu

**Affiliations:** Department of Immunization, Beijing Center for Disease Prevention and Control, No.16, HePingLi Middle Street, DongCheng District, Beijing, 100013 China

**Keywords:** Alcohol consumption, Alcoholic liver disease, Prevalence, Cross-sectional study, Risk factors

## Abstract

**Background:**

The alcohol consumption pattern, alcoholic liver disease (ALD) prevalence and related risk factors among alcohol drinkers in Beijing haven’t been fully elucidated. Hence, a cross-sectional study was conducted to investigate potential link among these factors.

**Methods:**

A two-stage stratified cluster sampling was carried out in Beijing. All participants were 25 years of age or older, possessed with medical insurance, and lived in Beijing for over 6 months. As part for this investigation, participants were asked to answer a questionnaire and undergo physical examination. The questionnaire included demographic information, alcohol intake, and medical history. The physical examination included physical and Fibrotouch tests. Moreover, 10 ml blood sample was collected from each subject to examine liver functions, perform routine blood, Hepatitis B Virus (HBV) and Hepatitis C Virus (HCV).

**Results:**

Overall, 74,988 residents participated in our study. The proportion of current drinkers among all participants was 46.10%. The differences in gender, region, age group, education, annual household income, and occupation among lifetime abstainers, former drinkers, non-weekly and weekly drinkers were significantly different (*P*<0.05). The ethanol intake between men and women, people living in urban and rural regions were significantly different (*P*<0.05). Strong spirits were commonly consumed by men, whereas, beers were commonly consumed by women. Drinking strong spirits generally lead to liver steatosis. In addition, ALD prevalence was 1.30% in participants over 25 years old. The differences in ALD prevalence between men and women, and among different age groups, were significant (*P*<0.05). Based on our analysis, ALD risk factors in Beijing included: gender (male), age (older than 35 years), high waist circumference, high blood pressure, high BMI, high blood sugar level, and being heavy drinkers.

**Conclusion:**

Compared with other cities or regions in China, the level of alcohol consumption in Beijing is at an upper middle level. But the ALD prevalence is low likely because ethanol intake is relatively low. Our analysis revealed that heavy drinking is a major risk factor for ALD development. Hence, if alcohol consumption is unavoidable, we caution against heavy drinking.

## Background

Chronic hepatitis B was once a dominant chronic liver disease in China, but the hepatitis B infection rate reduced significantly with the emergence of the hepatitis B vaccine in 1992 [1]. The prevalence of other chronic liver diseases, especially alcoholic liver disease (ALD) and nonalcoholic fatty liver disease (NAFLD), is rapidly increasing [2].

According to the 2018 World Health Organization (WHO) report on alcohol and health, alcohol consumption is estimated to result in 132.6 million disability-adjusted life years (DALYs) [3]. At the present time, mortality from alcohol consumption is higher than mortality caused by other diseases, such as tuberculosis, HIV/AIDS, and diabetes [3]. In China, production and consumption of alcoholic beverage has significantly increased due to a continuously growing economy [4]. Different alcoholic beverages contain varying levels of ethanol. Thus, it is imperative to collect a wide range of alcoholic beverages and analyze them separately. In the meantime, the number of ALD patients is rising at an alarming rate. Between 2006 and 2010, viral-hepatitis-related cirrhosis hospitalization declined by 10% in Beijing, but alcoholic cirrhosis-related hospitalization increased by 33%, according to a hospitalization summary report (HSR), based on the analysis of 2.3 million hospitalized patients in 31 Grad 3A hospitals [5]. The ratio of patients hospitalized with ALD to all hospitalized patients with liver diseases is rising almost continuously from 1.68% in 2002 to 4.59% in 2013, as reported by 302 hospital in Beijing [6].

Alcohol use disorder (AUD) is severe in China, especially in Northeast China [7]. Unfortunately, thus far, only region-based ALD studies, and not national epidemiological ALD survey, have been conducted in China [4, 8–14]. The point prevalence of ALD in certain regions in China is reported to range from 2.27–8.75% [15]. Unfortunately, the ALD prevalence in Beijing is currently unknown [15], as the latest national alcohol consumption survey was conducted 10 years ago in 2011 [16]. Meanwhile, some alcohol consumption studies, based on community residents, lacked a combined analysis involving ALD [17, 18]. Furthermore, ALD risk factor surveys in China yielded very different results, even opposite in studies that included the following factors: male gender, middle age, currently unmarried, education level, rural residence, family income, high level of occupation, high BMI, and smoking [4, 19]. Thus, we conducted an extensive study, involving community residents, to determine the prevalence of recent alcohol consumption, ALD prevalence of, and identify potential correlations between socio-economic, demographic, alcoholic consumption, and viral hepatitis infection with ALD in Beijing.

## Methods

### Subjects and sampling

Our work was part of an epidemiological study on the liver health of community residents in Beijing. It was based on a two-stage stratified cluster sampling carried out in 16 districts and 331 townships between 2017 and 2020. First, 11, out of 16 districts, were randomly sampled. Then, 2 townships in each district were randomly sampled. All residents within sampled townships were required to participate in the investigation. Each participant was asked to sign an informed consent agreement before survey. All participants were asked to answer a questionnaire, undergo physical examination, and blood sample collection. Each subject was then confirmed to ensure that the questionnaire, blood sampling, and physical examination were completed on the survey site. If any item was not completed, it was supplemented immediately. The inclusion criteria were as follows: (1) adults, aged 25 years and older; (2) residents who lived in Beijing for more than 6 months and possessed medical insurance. The exclusion criteria were as follows: (1) residents aged less than 25 years old; (2) residents with pacemakers; (3) pregnant women; (4) residents with a large amount of ascites; (5) residents with unhealed wound on the right upper abdomen that Fibrotouch test required.

Sampling size is estimated as:

$${\displaystyle \begin{array}{c}N=\frac{1.96^2\times P\times \left(1-P\right)}{\sigma^2}\times Deff\\ {} Deff=1+\left(M-1\right)\times ICC\\ {} ICC=\frac{K^2\times P}{1-P}\end{array}}$$

1.96 is the two-tailed Zα value where α is 0.05. P is the expected true proportion of ALD in Beijing, which is 5% in men and 3% in women [15]. σ is the relative precision, which is set to 0.02. K is the coefficient of between-cluster variation, which is set to 0.6. ICC is the intraclass correlation coefficient which is estimated to be 0.019 in men and 0.011 in women. M is the cluster size (number of targeted individuals), and it is approximately constant across clusters. The estimation of M is 5000 residents in each township. Deff corresponds to the design effect which is estimated to 95.7 in men and 56.6 in women. Considering the need to collect blood, the respondent rate is estimated to be 80%. The sample size in men is estimated to be 54,582. The sample size in women is estimated to be 19,794. The totol sample size is estimated to be 74,376.

### Questionnaire and physical examination

All investigators were trained prior to questionnaire and physical examination. Once participants signed the informed consent, a face-to-face questionnaire commenced. The questionnaire included: (1) demographic variables, included age, gender, region, education, occupation, nationality, marital and living status, and annual household income. (2) Evaluation of alcohol intake, included detailed questions on the use of alcoholic beverages, types of alcoholic beverage consumed, quantity of alcohol intake in each intake, the duration of drinking, and so on. (3) Medical history included prior diagnosis of chronic liver disease and other chronic diseases. The physical examination included the following: (1) height, weight, waist circumference, hip circumference, and blood pressure. (2) Fibrotouch test using the FibroTouch FT100 (WuXi Hisky Medical Technologies Co.Ltd), a new technology which indirectly assesses degree of liver fibrosis, similar to the FibroScan. Successful liver fibrosis measurement requires three conditions: (1) at least 10 valid measurements (2) 60% or higher success rate (3)inter-quartile range/median less than 33%. Those whose Fibrotouch DBM was≥240 db/m was diagnosed with liver steatosis. Those whose Fibrotouch LSM was≥12.96 Kpa was diagnosed with liver fibrosis.

### Blood collection and assessment

10 ml blood sample was collected from each participants to examine liver function, routine blood, HBsAg, anti- HBsAg, anti- HBc, and anti- HCV. The anti-HCV positive participants were further tested for HCV RNA. All liver function (ALT, AST, GGT) examinations were performed on the Hitachi 7600–110 automatic analyzer (Hitachi High-Technologies, Tokyo, Japan), using reagents from Wako (Pure Chemical Industry, Japan). Routine blood tests were analyzed with the Cell-DYN Ruby (Abbott Laboratories, Diagnostic Division, Abbott Park, IL, USA) within 2 h of collection. HBV serological markers were tested by Architect i2000 (Chemiluminescence MicroparticleImunoassay, Abbott, Chicago, USA). HCV serological markers were initially tested using the Colloidal Gold method (YingkeXinChuang, China), followed by confirmation of the positive anti-HCV samples were confirmed with an enzyme immune assay (ARCHITECT Anti-HCV; Abbott Laboratories, USA). The anti-HCV positive samples were tested for HCV RNA using the Abbott Real Time HCV (Abbott Laboratories), carrying a sensitivity of 15 IU/ml for the determination of viral load.

### Alcohol consumption assessment

The participants who consumed alcoholic beverages in the past12-month period were classified as current drinkers. The current drinkers were subcategorized into non-weekly and weekly drinkers. The current drinkers who consumed alcohol occasionally (less than weekly) were classified as non-weekly drinkers. The current drinkers who drank alcohol at least once a week were classified as weekly drinkers. Those who never consumed alcohol were classified as lifetime abstainers. Those who previously consumed alcohol, but has not had a drink in the past12-month period were classified as former drinkers. The aforementioned drinking categories were in line with the WHO global status report on alcohol and health 2018 [3], and WHO guide for monitoring alcohol consumption [20].

The current drinkers who consumed weekly in the past 12 months were further asked questions on types of beverage (beer, grape wine, rice wine, weak spirits < 40% alcohol content, or strong spirits ≥40% alcohol content), numbers of alcohol intake per week, and amount of alcohol consumed each time (reported by number of small (330 ml) or large (640 ml) bottles for beer, and number of liang (50 g) for wines and spirits). The level of alcohol consumption was calculated as grams of ethanol per day, based on the beverage type, amount consumed, and consumption frequency per week. Our analysis revealed the following alcohol content by volume (v/v) in China: beer 4%, grape wine 12%, rice wine 15%, weak spirits 38%, and strong spirits 53%. Grams of ethanol per day = [Beer consumption frequency per week × numbers of (small bottles 330 ml or large bottles 640 ml) each time× 4% × 0.8/7] + [grape wine consumption frequency per week × number of liang (50 g) each time × 12% × 0.8/7] + [rice wine consumption frequency per week × number of liang (50 g) each time× 15% × 0.8/7] + [weak spirits consumption frequency per week × number of liang (50 g) each time× 38% × 0.8/7] + [strong spirits consumption frequency per week × number of liang (50 g) each time × 53% × 0.8/7] [3]. Heavy drinkers were those who consumed more than 60 g of ethanol per day.

### Diagnostic criteria

ALD diagnosis was confirmed according to the Guidelines of Prevention and Treatment for Alcoholic Liver Disease: a 2018 updated version drafted by the National Workshop on Fatty Liver and Alcoholic Liver Disease, Chinese Society of Hepatology Chinese Medical Association and Fatty Liver Expert Committee, and Chinese Medical Doctor Association [21]. ALD diagnosis met all of the following criteria: (1) men who consumed more than 40 g of ethanol per day or women who consumed more than 20 g of ethanol per day; (2) those who consumed alcohol for more than 5 years; (3) [(ALT> 40 U/L or AST > 40 U/L) and AST/ALT> 2] or GGT > 55 U/L or MCV > 96 fL; (4) those with liver steatosis (Fibrotouch DBM ≥ 240 db/m) or liver fibrosis (Fibrotouch LSM ≥ 12.96 Kpa); (5) those with HBV or HCV infection were excluded (positive of HBsAg or HCV RNA) .

### Statistical analysis

Epidata (3.1) was used to establish databases. All participant information was entered separately by two groups from the Beijing XunChiFeiLong Data Technology Co.Ltd. Data check was performed independently by two investigators from the same company. Data were analyzed using the statistical analysis package SPSS (SPSS Inc., Chicago IL, version 19.0). Differences in categorical variables were tested using the *χ*^*2*^ or Fisher’s exact test. Continuous variables, with normal distribution, are presented as means± standard deviation (SD), and the differences were tested by Student’s t-test or Anova test. A *p*-value< 0.05 (2-tailed) was considered statistically significant. Considering the potential strata clustering (geographical, urban/rural areas) effect on the ALD prevalence, we used a linear mixed model to do the multilevel analysis first. However, we did not observe the significant effect of group level. So, we employed multivariable logistic regression models to examine the factors associated with ALD. All factors for which the *P*-value of univariate analysis was < 0.05 were entered into the model. The stepwise regression method was used and *p*-value< 0.05 was considered significant. Adjusted odd ratios (OR) and 95% confidential intervals, derived from a logistic-regression model, were used to assess the relationships between ALD and socio-economics, demographic characteristics, and alcoholic consumption variables.

## Results

### Socio-demographic characteristics of subjects

Seventy-six thousand two hundred twenty residents in 22 townships were asked to participate in our survey, and 74,988 residents completed the questionnaire, physical examination, and blood collection (Table 1). The response rate was 98.40%. Among them, 40,148 participants were lifetime abstainers; 278 participants were former drinkers; 20,972 participants were non-weekly drinkers; and 13,600 participants were weekly drinkers. The proportion of current drinkers among all participants was 46.10%. The initial drinking age was 25.53 ± 9.47 years old. The differences in gender, regional, age group, educational, annual household income distribution, and occupational distributions among the lifetime abstainers, former drinkers, non-weekly and weekly drinkers were statistically significant (*P*<0.05). There were more lifetime abstainers among women, and more current drinkers among men. The proportion of lifetime abstainers increased with age, but there was an opposite trend among current drinkers. The proportion of lifetime abstainers decreased with the increase of education level and annual household income, but an opposite trend was seen among current drinkers. The grams of ethanol intake per day, among weekly drinkers, between men and women, were significantly different (*P*<0.05). It was confirmed that the grams of ethanol intake per day among male weekly drinkers were higher than among females. Moreover, the grams of ethanol intake per day among weekly drinkers, between people living in urban and rural areas, were statistically different as well (*P*<0.05). Interestingly, the grams of ethanol intake per day among weekly drinkers in rural area were higher than those living in urban area. The grams of ethanol intake per day among heavy drinkers, between men and women, urban and rural residents, however, was not statistically different (*P* > 0.05).

**Table 1:** Prevalence of alcohol consumption and grams of ethanol per day, by socio-demographic characteristics

	Total	Lifetime Abstainer N(%)	Former Drinker N(%)	Current Drinker					***χ***^***2***^
				Non-weekly N(%)	Weekly				
					N(%)	Alcoholintake^**a**^	The age began to drink	Alcoholintake in heavy drinker^**b**^	
**All**	74,988	40,148(53.53)	278(0.37)	20,972(27.96)	13,600(18.13)	37.18 ± 40.13	25.53 ± 9.47	98.47 ± 51.77	
**Gender**									*χ*^*2*^ = 21,467.15 *P* = 0.000
Men	34,567	9237(26.72)	198(0.57)	12,791(37.00)	12,341(35.70)	39.21 ± 40.78	24.82 ± 8.76	98.52 ± 51.80	
Women	40,431	30,911(76.45)	80(0.20)	8181(20.23)	1259(3.11)	15.81 ± 23.42	33.04 ± 12.83	90.41 ± 51.62	
**Region**	74,998	40,148(53.53)	278(0.37)	20,972(27.96)	13,600(18.13)				*χ*^*2*^ = 581.61 *P* = 0.000
Urban	41,999	22,288(53.07)	147(0.35)	12,946(30.82)	6618(15.76)	36.05 ± 38.92	25.79 ± 9.63	98.90 ± 50.35	
Rural	32,999	17,860(54.12)	131(0.40)	8026(24.32)	6982(21.16)	38.23 ± 41.18	25.29 ± 9.32	98.11 ± 52.95	
**Age Group**									*χ*^*2*^ = 2790.18 *P* = 0.000
25~	9958	4794(48.14)	46(0.46)	3979(39.96)	1139(11.44)	26.14 ± 32.44	20.61 ± 3.63	102.89 ± 49.22	
30~	8513	4087(48.01)	31(0.36)	3201(37.60)	1194(14.03)	30.11 ± 42.36	22.18 ± 4.26	104.00 ± 81.05	
35~	8292	4099(49.43)	13(0.16)	2876(34.68)	1304(15.73)	32.51 ± 37.69	23.12 ± 5.36	103.02 ± 50.20	
40~	6664	3412(51.20)	25(0.38)	2046(30.70)	1181(17.72)	32.50 ± 38.63	24.23 ± 6.73	98.06 ± 54.48	
45~	7098	3774(53.17)	25(0.35)	1941(27.35)	1358(19.13)	38.53 ± 38.32	25.07 ± 8.23	95.35 ± 41.01	
50~	9529	5035(52.84)	32(0.34)	2254(23.65)	2208(23.17)	41.18 ± 39.94	26.16 ± 9.00	97.54 ± 44.90	
55~	6887	3811(55.34)	25(0.36)	1476(21.43)	1575(22.87)	43.84 ± 43.72	26.39 ± 9.70	100.66 ± 50.84	
60~	18,057	11,136(61.67)	81(0.45)	3199(17.72)	3641(20.16)	39.93 ± 40.57	28.72 ± 12.56	96.49 ± 53.48	
**Education**									*χ*^*2*^ = 3219.11 *P* = 0.000
illiteracy	1510	1127(74.64)	4(0.26)	159(10.53)	220(14.57)	39.91 ± 37.58	30.51 ± 14.20	93.61 ± 40.35	
Primary school	5856	3851(65.76)	31(0.53)	812(13.87)	1162(19.84)	40.72 ± 42.06	27.35 ± 12.07	97.77 ± 48.21	
Junior middle school	24,107	13,248(54.95)	91(0.38)	5257(21.81)	5511(22.86)	39.58 ± 40.20	26.0 ± 9.88	97.19 ± 49.09	
High school	18,281	9603(52.53)	65(0.36)	5107(27.94)	3506(19.18)	36.68 ± 40.38	24.95 ± 8.73	99.82 ± 53.97	
Graduate	22,577	11,158(49.42)	85(0.38)	8315(36.83)	3019(13.37)	30.22 ± 38.73	24.21 ± 7.53	101.33 ± 59.37	
Postgraduate	2667	1161(43.53)	2(0.07)	1322(49.57)	182(6.81)	23.65 ± 30.18	25.49 ± 7.20	93.66 ± 42.24	
**Annual household income**									*χ*^*2*^ = 1830.90 *P* = 0.000
< 4000$	14,938	9082	74	2504	3278	43.29.21 ± 44.61	26.69 ± 10.83	100.45 ± 55.76	
4000$-20,000$	45,845	24,571	154	12,926	8194	36.81 ± 38.95	25.25 ± 9.17	96.93 ± 49.35	
20,000$-40,000$	12,083	5593	45	4619	1826	33.97 ± 39.81	24.78 ± 8.13	103.83 ± 55.29	
≥ 40,000$	2132	902	5	923	302	38.45 ± 38.66	24.97 ± 7.53	91.43 ± 39.24	
**Occupation**									*χ*^*2*^ = 3859.61 *P* = 0.000
Government Employee	8778	4160(47.39)	30(0.34)	3127(35.62)	1461(16.61)	36.18 ± 40.86	24.07 ± 6.88	101.38 ± 51.16	
Corporate Employee	12,156	6290(51.74)	54(0.44)	3376(27.77)	2436(20.04)	35.89 ± 40.32	24.37 ± 7.81	99.22 ± 57.23	
Teacher & Institute Employee	10,144	4667(46.01)	35(0.35)	4085(40.27)	1357(13.38)	29.57 ± 32.71	24.03 ± 6.92	95.02 ± 46.57	
Urban or rural worker	16,504	8362(50.67)	56(0.34)	4443(26.92)	3643(22.07)	36.03 ± 39.03	25.79 ± 9.89	96.39 ± 51.22	
Retiree	9487	5950(62.72)	31(0.33)	1953(20.59)	1553(16.37)	39.45 ± 40.41	29.03 ± 12.93	96.92 ± 55.28	
Other	17,929	10,719(59.79)	72(0.40)	3988(22.24)	3150(17.57)	41.98 ± 42.78	25.70 ± 9.60	100.22 ± 48.87	

^a^Alcohol_intake assessed by grams of ethanol per day (grams/day)

^b^Heavy drinker: those who consum more then 60 g of ethanol per day

### Types of alcohol and consumption

The alcohol consumption of different alcoholic beverages among weekly drinkers was significantly different (*P*<0.05). Strong spirit consumption was the highest among weekly drinkers, which was up to 49.88%. The second and third alcohol consumption was related to weak spirits and beer consumption (Table 2). Those who drank strong spirits consumed more than twice as much ethanol as those who drank beer. Strong spirits were commonly consumed by men, whose ethanol intake was also the highest. In contrast, beer consumption was common among women. In addition, the ethanol intake in men was higher than in women, due to the consumption of different types of alcohol beverage. People in urban areas preferred strong spirits and beer (F = 106.45, *P*<0.05), whereas, rural residents preferred weak and strong spirits(F = 167.05, *P*<0.05). Although people in urban areas preferred strong spirits, the ethanol intake were lower than their rural counterparts(*P*<0.05).

**Table 2:** Consumption of different alcoholic beverages among weekly drinkers

		Beer			Grape wine			Rice wine			Weak spirits			Strong spirits		
	N	%	Alcoholintake^**a**^	T value	%	Alcoholintake^**a**^	T value	%	Alcoholintake^**a**^	T value	%	Alcoholintake^**a**^	T value	%	Alcoholintake^**a**^	T value
**Total**	13,600	34.69	15.84 ± 22.38		5.38	4.02 ± 4.27		1.29	5.94 ± 8.58		42.71	24.04 ± 21.25		49.88	42.91 ± 38.45	
**Gender**				t = 16.12 *P*<0.05			t = 3.76 *P*<0.05			t = 3.10 *P*<0.05			t = 19.18*P*<0.05			t = 9.80 *P*<0.05
Men	12,341	34.03	16.82 ± 12.29		3.33	4.51 ± 4.86		1.13	6.62 ± 9.21		43.31	25.03 ± 21.57		52.49	43.73 ± 38.56	
Women	1259	41.14	7.86± 9.65		25.50	3.38 ± 3.27		2.86	3.24 ± 4.53		36.85	12.64 ± 12.37		24.31	25.48 ± 31.44	
**Region**				t = 0.03 *P*>0.05			t = −1.46 *P*>0.05			t = −0.33 *P*>0.05			t = −6.53 *P*<0.05			t = −6.93 *P*<0.05
Urban	6618	38.18	15.85 ± 21.02		7.71	3.86 ± 4.21		1.87	5.80 ± 9.35		37.29	21.93 ± 19.83		53.99	39.84 ± 37.43	
Rural	6982	31.38	15.83 ± 23.85		7.95	4.36 ± 4.39		0.74	6.27 ± 6.48		47.85	25.60 ± 22.12		45.99	46.32 ± 39.27	

^a^Alcohol_intake assessed by grams of ethanol per day (g/d)

### The relationship between types of alcohol, consumption, liver steatosis, and fibrosis

Increased ethanol intake and alcohol content in beer, grape wine, rice wine, weak spirit, and strong spirit significantly correlated with severity of liver steatosis (*P*<0.05). However, no such correlation was observed with the severity of liver fibrosis (*P* > 0.05). Hence, excessive spirit consumption and ethanol intake dramatically increased the probability of developing liver steatosis (Table 3).

**Table 3:** The relationship between types of alcohol, consumption, liver steatosis, and fibrosis

	N	Liver steatosis (%)		***χ***^***2***^	Liver fibrosis (%)		***χ***^***2***^
		< 240	≥240		< 12.96	≥12.96	
**Alcoholintake**				*χ*^*2*^ = 10.23 *P* = 0.02			*χ*^*2*^ = 1.84 *P* = 0.60
< 20	5597	3308(59.10)	2289(40.92)		5450(97.37)	147(2.63)	
20~	3233	1897(58.68)	1336(41.32)		3145(97.28)	88(2.72)	
40~	2163	1252(57.88)	911(42.12)		2104(97.27)	59(2.73)	
≥ 60	2607	1446(55.47)	1161(44.53)		2525(96.75)	82(3.15)	
**Types of alcohol**				*χ*^*2*^ = 29.72 *P* = 0.00			*χ*^*2*^ = 8.75 *P* = 0.06
Beer	1849	1145(61.93)	704(38.07)		1805(97.62)	44(2.38)	
Grape wine	348	234(67.24)	114(32.76)		345(99.14)	3(0.86)	
Rice wine	51	35(68.63)	16(31.37)		51(100.00)	0(0.00)	
Weak spirits	4565	2596(56.87)	1969(43.13)		4424(96.91)	141(3.09)	
Strong spirits	6784	3892(57.37)	2892(42.63)		6596(97.23)	188(2.77)	

### Related examination and prevalence of ALD among weekly drinkers

**Fig. 1:**
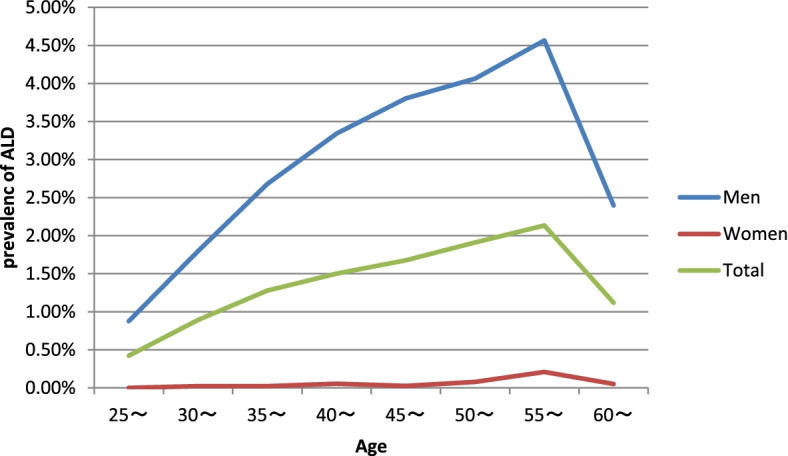
ALD prevalence among men and women, according to age

Among the 13,600 weekly drinkers, 4934 participants (36.28%) were men who consumed more than 40 g of ethanol per day, or women who consumed more than 20 g of ethanol per day. In either case, both consumed alcohol for more than 5 years. In addition, 618 participants (4.54%) were ALT> 40 U/L or AST > 40 U/L and AST/ALT> 2, 3726 participants (27.40%) were GGT > 55 U/L, 270 participants (1.99%) were MCV > 96 fL, 5697 participants (41.89%) had liver steatosis (Fibrotouch DBM ≥ 240 db/m), 376 participants (2.76%) had liver fibrosis (Fibrotouch LSM ≥ 12.96 Kpa), and 974 participants were diagnosed with ALD. Multilevel analysis finds the different districts, and rural or urban areas have no effect on the residents’ALD prevalence (P>0.05). The proportion of ALD among weekly drinkers was 7.16% (974/13600), among adults over 25 years old was 1.30% (974/74988), among men was 2.75% (952/34567), among women was 0.05% (22/40431). The proportion of ALD among weekly drinkers between men and women (*χ*^*2*^ = 61.17, *P*<0.05) and among age groups (*χ*^*2*^ = 61.23, *P*<0.05) were statistically significant. ALD in men was higher than in women, and the highest incidence was in people aged between 50 and 60 years old (Fig. 1). The proportion of ALD in urban and rural areas was not statistically significant (*P*>0.05). The proportion of ALD among residents in urban areas was 1.19%(501/41999), among residents in rural areas was 1.43%(473/32999).

### Influence and risk factors for ALD

ALD diagnosis was used as a dependent variable. The independent variables used to conduct logistic regression analysis were: gender, age group, BMI, waist circumference, hip circumference, blood pressure, occupation, education, marital status, annual household income, heavy drinker or not, and blood sugar level. The final analysis presented in Table 4. Gender (male), age (older than 35 years old), increased waist circumference, high blood pressure (systolic pressure ≥ 140 mmHg or diastolic pressure ≥ 90 mmHg), high BMI, high blood sugar level, and being heavy drinkers were risk factors for ALD. Increased hip circumference was a protective factor for ALD.

**Table 4:** Logistic regression analyses of influence and risk factors for ALD

Variables	*β*	Standard error	P	OR(95% CI)
Gender (basedon female)	0.68	0.23	0.00	1.97(1.25–3.10)
Age group (Based on 25–29)				
25~	–	–	–	1
30~	0.25	0.23	0.28	1.29(0.82–2.03)
35~	0.47	0.19	0.02	1.60(1.09–2.33)
40~	0.61	0.18	0.00	1.84(1.30–2.59)
45~	0.56	0.17	0.00	1.74(1.25–2.44)
50~	0.39	0.16	0.02	1.48(1.08–2.04)
55~	0.36	0.15	0.01	1.44(1.09–1.91)
60~	0.51	0.15	0.00	1.66(1.24–2.22)
Waist circumference	0.06	0.01	0.00	1.06(1.04–1.07)
Hip circumference	−0.04	0.01	0.00	0.96(0.94–0.98)
HBP (High Blood Pressure)	0.39	0.08	0.00	1.48(1.27–1.74)
BMI	0.14	0.02	0.00	1.15(1.12–1.19)
Blood sugar	0.06	0.02	0.00	1.06(1.03–1.09)
Heavydrink	2.07	0.08	0.00	7.90(6.81–9.17)

### Comparison between heavy drinkers and non-heavy drinkers according to ALD

Among the non-ALD participants, the differences between heavy and non-heavy drinkers in terms of age, waist circumference, hip circumference, BMI, systolic BP, diastolic BP, blood sugar, total cholesterol, HDL, AST and DBM were statistically significant. Among the ALD participants, the differences between heavy and non-heavy drinker in terms of only HDL and LDL were statistically significant. However, the difference in terms of age, waist circumference, hip circumference, BMI, systolic BP, diastolic BP, blood sugar, total cholesterol, AST and DBM were not statistically significant. There was found more diagnosed with high HDL and LDL among heavy drinkers than non-heavy drinkers in ALD participants (Table 5). This suggested that the liver function was affected by heavy ethanol intake, even in current drinkers who were not diagnosed with ALD. However, for ALD patients whose liver function were impaired, heavy ethanol intake did not worsen liver condition, which is in accordance with the ceiling effect. These evidences suggested that current drinkers not yet diagnosed with ALD must avoid heavy ethanol intake in order to protect their livers.

**Table 5:** Comparison of heavy drinkers versus non-heavy drinkers, according to ALD

	Non-ALD			ALD		
	Non-heavy drinker	Heavy drinker	T	Non-heavy drinker	Heavy drinker	T
	*N* = 10,604	*N* = 2022		*N* = 390	*N* = 584	
Age	49.02 ± 13.64	53.00 ± 11.51	t = 13.80 *P* = 0.00	49.45 ± 11.06	49.45 ± 11.40	t = −0.01 *P* = 0.996
Waist circumference (cm)	89.86 ± 10.04	90.36 ± 9.10	t = 2.21 *P* = 0.03	97.43 ± 8.33	97.82 ± 8.13	t = 0.73 *P* = 0.46
Hip circumference (cm)	99.49 ± 7.38	99.17 ± 6.73	t = −1.99 *P* = 0.047	103.52 ± 6.85	103.22 ± 6.60	t = −0.68 *P* = 0.49
BMI	25.93 ± 3.69	25.63 ± 3.35	t = −3.69 *P* = 0.00	28.83 ± 3.05	28.64 ± 3.45	t = −0.90 *P* = 0.37
Systolic BP (mmHg)	135.84 ± 20.22	141.17 ± 20.76	t = 10.82 *P* = 0.00	143.88 ± 20.83	143.40 ± 19.54	t = − 0.37 *P* = 0.71
Diastolic BP (mmHg)	82.88 ± 12.98	84.58 ± 13.33	t = 5.36 *P* = 0.00	88.14 ± 13.48	88.30 ± 13.27	t = 0.18 *P* = 0.86
Blood sugar (mmol/L)	6.14 ± 1.87	6.32 ± 2.04	t = 3.73 *P* = 0.00	6.88 ± 2.32	6.72 ± 1.99	t = −1.07 *P* = 0.29
Total cholesterol (mmol/L)	5.14 ± 1.01	5.34 ± 1.07	t = 7.89 *P* = 0.00	5.60 ± 1.21	5.74 ± 1.31	t = 1.76 *P* = 0.08
HDL (mmol/L)	1.37 ± 0.35	1.50 ± 0.41	t = 13.08 *P* = 0.00	1.31 ± 0.33	1.36 ± 0.37	t = 2.49 *P* = 0.01
LDL (mmol/L)	3.05 ± 1.76	3.07 ± 1.91	t = 0.42 *P* = 0.67	3.12 ± 0.96	3.27 ± 0.99	t = 2.22 *P* = 0.03
Triglyceride (mmol/L)	12.20 ± 298.55	12.25 ± 270.17	t = 0.01 *P* = 0.995	3.60 ± 3.80	3.50 ± 3.65	t = −0.39 *P* = 0.70
ALT (U/L)	24.80 ± 19.85	23.80 ± 21.84	t = −1.90 *P* = 0.06	39.05 ± 26.14	39.29 ± 28.18	t = 0.13 *P* = 0.89
AST (U/L)	23.69 ± 13.76	25.89 ± 22.29	t = 4.29 *P* = 0.00	33.28 ± 26.09	34.19 ± 23.03	t = 0.58 *P* = 0.56
DBM (db/m)	234.41 ± 37.38	228.75 ± 32.81	t = −6.95 *P* = 0.00	270.46 ± 25.99	270.06 ± 25.48	t = −0.24 *P* = 0.81
LSM (Kpa)	6.86 ± 3.38	6.78 ± 2.97	t = −1.08 *P* = 0.28	8.08 ± 4.16	8.24 ± 4.32	t = 0.58 *P* = 0.56

## Discussion

This study is one of the largest surveys on liver health among community residents in Beijing this year. In this population-based study, alcohol consumption, the ALD prevalence and correlation of socio-economics, demographic characteristics, alcohol consumption, and viral hepatitis infection with ALD were investigated.

The proportion of lifetime abstainers, former drinkers, and current drinkers among residents older than 25 years of age were 53.53, 0.37, and 46.10% respectively. Almost half of the community residents were current drinkers, which was a considerably large number. The amount of ethanol intake in weekly drinkers was 37.18 ± 40.13 g/day, while among heavy drinkers, it reached 98.47 ± 51.77 g/day. The initial drinking age was 25.53 ± 9.47 years old. Alcohol consumption was much more frequent among men than women, and it was more frequent in rural areas than urban areas. Similar to other areas in China, men consumed more alcohol than women [22]. The reasons for high alcohol consumption in men may include: more opportunities to participate in social activities and expose themselves to environments of alcohol abuse, and men don’t have a strong sense of self-protection due to traditional education, and thus increase their alcohol intake. In rural areas, people work very hard during the day, so they tend to drink more wine to relieve fatigue at night. The amount of ethanol intake in men and rural areas were also higher than in women and urban regions. Younger age, higher education level and more household income led to a higher proportion of alcohol consumption. These data reflect the relationship between alcohol consumption, geography, economics, and culture. The alcohol consumption varied among different areas and provinces. In a 2019 review [15], the percentage of regular alcohol drinkers, among general Chinese adults in different areas, was shown to be the lowest at 27% in Zhengjiang and the highest at 66.2% in Shanxi, Gansu, and Xinjiang. Compared to other cities in this review, we revealed that the alcohol consumption in Beijing was at an upper middle level. Strong spirit consumption among weekly drinkers was the highest. Strong spirit was commonly consumed by men, and their ethanol intake was also the highest. In contrast, beers were commonly consumed by women. Drinking strong spirit and more ethanol intake were more likely to lead to liver steatosis, but such correlation was not identified with liver fibrosis. Although people living in urban areas preferred strong spirits, their ethanol intake was lower than people living in rural areas. It was also found that the ethanol intake of Beijing residents in different wines (strong spirits, weak spirits, beer, rice wine and grape wine) were lower than ten other provinces in China [17].

In this study, we observed that the ALD prevalence was 1.30% among permanent residents and 7.16% among weekly drinkers. The ALD prevalence in Beijing was lower than other provinces, which was between 2.27–8.74% [8–14]. The proportion of men who consumed more than 40 g of ethanol per day or women who consumed more than 20 g of ethanol per day to weekly drinkers was 36.28%, which meant that ethanol intake among most weekly drinkers was low, and not at an abusive level. This may be because the economy level in Beijing is high, and most people are able to detect illness in advance via physical examinations, therefore, tend to abstain from alcohol or drink less. The ALD prevalence in urban and rural areas was not statistically significant. The ALD prevalence in men was 2.75%, while in women it was 0.05%. The higher ALD prevalence in men is in accordance with other studies in China [8–14]. Meanwhile, the ALD prevalence was the highest among individuals aged between 50 and 60 years old. With rise in the participants’ age, the number of current drinks went down. However, the amount of ethanol intake increased, which may result in a higher ALD prevalence in the elderly. Such correlation is different from what was reported in foreign studies, in which the highest ALD prevalence was usually found in young residents aged between 18 and 34 years old [4]. In our study, with increasing age, the ethanol intake increased, which revealed that the young people in China typically drink alcoholic beverages containing low ethanol, such as beer. This led to a higher proportion of young people drinking alcohols, but with low ethanol intake, and therefore, failure to meet the ALD diagnostic criteria. In addition, the Chinese tradition does not encourage young people to consume alcohol. Hence, only middle-aged and older participants participate in social activities. These individuals likely have a stable family income, so they have more access to alcoholic beverages. All these factors contribute to the higher prevalence of ALD in China among individuals between 50 and 60 years old.

Several factors regulate ALD occurrence [23, 24]. In this study, gender (male), age (older than 35 years old), increased waist circumference, high blood pressure, high BMI, high blood sugar level, and being heavy drinkers were risk factors for ALD in Beijing. These risk factors for ALD are the same as other studies [15, 23, 24]. Being a heavy drinker is a very serious risk factor for ALD and the OR is up to 7.90. For current drinkers who were not diagnosed with ALD, their liver function was affected by heavy ethanol intake. But for ALD patients whose liver function were impaired, heavy ethanol intake did not worsen their liver condition, which is in accordance with the ceiling effect. These evidence suggested that if alcohol intake cannot be avoided, drinking heavily should strictly be avoided.

Although this study fills the void in data on the ALD prevalence in Beijing, there are still some limitations that deserve further discussion. First, this study was only conducted in Beijing and does not cover other regions. Therefore, we can’t infer the ALD prevalence in the whole country using this study. Second, this was a cross-sectional study and it lacks effective follow-ups to observe the health condition of residents. Causal relationship cannot be examined based on cross-sectional data. Third, a major limitation of all alcohol epidemiology is that the exposure is uncertain. The main reason is recall bias. Drinking patterns are variable, and intake may be substantially underperceived or underreported. Despite these limitations, the sample size of this study was considerably large, and could fill the void of alcohol usage and ALD prevalence in Beijing.

## Conclusions

The level of alcohol consumption in Beijing was at an upper middle level compared with other cities or regions in China. There is a higher proportion of alcohol drinkers in male residents, people living in rural areas, younger people, people who have received higher education, and people with higher income. Strong spirits are commonly consumed by men, while beers are commonly consumed by women. Strong spirit and heavy ethanol intake are more likely to lead to liver steatosis. Although half of the residents are current drinkers, the prevalence of ALD is at a low level, because the amount of ethanol intake are not high in Beijing. There is a higher prevalence of ALD in men and in individuals aged from 50 to 60 years old. Being a heavy drinker is a major risk factor for ALD and should be avoided. if alcohol consumption is unavoidable, we caution against heavy drinking.

## Data Availability

The data sets used and/or analyzed for the current study are available from the corresponding author upon reasonable request**.**

## Abbreviations

AUD: Alcohol use disorder
ALD: Alcoholic liver disease
NAFLD: Nonalcoholic fatty liver disease
HBV: Hepatitis B virus
HCV: Hepatitis C virus
WHO: World Health Organization
DALYs: Disability-adjusted life years
HIV: Human immunodeficiency virus
AIDS: Acquired Immune Deficiency Syndrome
HSR: Hospitalization summary report
OR: Odd ratios
